# Supplementary material for: Microalgae Encapsulation Systems for Food, Pharmaceutical and Cosmetics Applications
Source: Mar Drugs. 2020 Dec 15;18(12):644. doi: 10.3390/md18120644 (PMC7765346; doi:10.3390/md18120644)
Supplement: Supplementary file 1 [file marinedrugs-18-00644-s001.pdf]

Supplementary Material: Microalgae encapsulation systems for food, pharmaceutical and cosmetics applications

Marta V. Vieira, Lorenzo M. Pastrana and Pablo Fuciños

Table S1. Different coating materials used for encapsulation systems and their CAS number.

Classification	Type of material	Compound	CAS number
Natural resources	Carbohydrates	Alginate	9005-38-3
		Maltodextrin	9050-36-6
		Pectin	9000-69-5
		Cellulose	9004-34-6
		Inulin	9005-80-5
		Chitosan	9012-76-4
	Proteins	Gelatin	9000-70-8
		Whey protein	91082-88-1
		Casein	9000-71-9
		Bovine Serum Albumin	9048-46-8
	Gums	Arabic	9000-01-5
		Guar	9000-30-0
Synthetic polymers	Biodegradable	Poly (ε-caprolactone)(PCL)	24980-41-4
		Poly (lactic acid) (PLA)	26100-51-6
		Poly (lactic-co-glycolic acid) (PLGA)	26780-50-7
	Non-biodegradable	Carboxymethyl cellulose	9004-32-4
		Cellulose acetate	9004-35-7
		Polyvinyl alcohol (PVA)	9002-89-5
